# Supplementary material for: Molecular characterization of renal cell carcinoma tumors from a phase III anti-angiogenic adjuvant therapy trial
Source: Nat Commun. 2022 Oct 10;13:5959. doi: 10.1038/s41467-022-33555-8 (PMC9550765; doi:10.1038/s41467-022-33555-8)
Supplement: Supplementary file 1 — Supplementary Tables and Figures [file 41467_2022_33555_MOESM1_ESM.pdf]

Supplementary Table 1 Patient demographics and baseline characteristics.

	WES Population			GES Population		
**Variables, n (%)**	**Sunitinib n=91**	**Placebo n=80**	**P-value**	**Sunitinib n=72**	**Placebo n=61**	**P-value**
Age, years						
<65	67 (73.6)	52 (65.0)	0.2461	53 (73.6)	43 (70.5)	0.7025
≥65	24 (26.4)	28 (35.0)		19 (26.4)	18 (29.5)	
Race						
White	76 (83.5)	70 (87.5)	0.3160	58 (80.6)	53 (86.9)	0.3002
Black	0	1 (1.3)		0	1 (1.6)	
Asian	13 (14.3)	6 (7.5)		12 (16.7)	5 (8.2)	
Other	2 (2.2)	3 (3.8)		2 (2.8)	2 (3.3)	
ECOG PS						
0	65 (71.4)	59 (73.8)	1.000	50 (69.4)	40 (65.6)	0.8497
1	24 (26.4)	21 (26.3)		20 (27.8)	20 (32.8)	
2	1 (1.1)	0		1 (1.4)	0	
NR	1 (1.1)	0		1 (1.4)	1 (1.6)	
UISS high-risk group						
T3 low[a]	28 (30.8)	25 (31.3)	0.9623	22 (30.6)	20 (32.8)	0.3884
T3 high[b]	56 (61.5)	48 (60.0)		47 (65.3)	36 (59.0)	
T4 N0 or NX, M0[c]	2 (2.2)	1 (1.3)		1 (1.4)	0	
Any T, N1-2, M0[c]	5 (5.5)	6 (7.5)		2 (2.8)	5 (8.2)	

[a] N0 or NX, M0, any Fuhrman's grade and ECOG PS 0 or T3 N0 or NX, M0, Fuhrman's grade 1 and ECOG PS ≥1.
[b] N0 or NX, M0, Fuhrman's grade ≥2 and ECOG PS ≥1.
[c] Any Furhman's grade and any ECOG PS.
Race, height, and weight were recorded at each site during screening. Race was investigator-reported in the study Clinical Research Form.
A two-sided log-rank test was used to obtain the P-values; no adjustments were made for multiple comparisons
ECOG PS=Eastern Cooperative Oncology Group performance status; GES=gene expression signatures; M=metastasis; N=node; NR=not reported; NX=nearby lymph nodes cannot be assessed due to lack of information; T=tumor; UISS=University of California-Los Angeles Integrated Staging System; WES=whole exome signatures

Supplementary Table 2 Complete listing of each institutional review board or independent ethics committee, listed by country

**Czech Republic**
Multicentricka eticka komise Fakultni nemocnice Motol<br>V Uvalu 84<br>Praha 5, CZECH REPUBLIC 150 06
**France**
Centre Hospitalier Universitaire Pontchaillou<br>Comite de protection des personnes "Ouest V"<br>Pavillon Clemenceau<br>Rennes cedex, FRANCE 35033
**Germany**
Ethik-Kommission der Medizinischen Fakultaet der Ludwig-Maximilians Universitaet Muenchen<br>Pettenkoferstr. 8a<br>Muenchen, GERMANY 80336
**Greece**
National Ethics Committee<br>284 Mesogeion Avenue Cholargos<br>Athens, ATTIKI GREECE 15562
**Ireland**
SJH/AMNCH Research Ethic Committee<br>FDVH Annex Adelaide and Meath Hospital Tallaght<br>Dublin, IRELAND 24
**Italy**
Comitato Etico A.O.R.N. A. Cardarelli A.O.R.N. Santobono-Pausilipon<br>Via Antonio Cardarelli, 9<br>Napoli, ITALY 80131
Comitato Etico Centrale IRCCS Lombardia,<br>Fondazione IRCCS Instituto Nazionale dei Tumori di Milano<br>Via Venezian, 1<br>Milano, ITALY 20133
Comitato Etico Delle provincie di Chieti e Pescara ASL di Lanciano -Vasto- Chieti<br>Via dei Vestini 29B<br>Chieti Scalo, ITALY 66013
Comitato Etico Indipendente dell'Azienda Ospedaliero Universitaria,<br>Policlinico Sant' Orsola<br>Padiglione 3 Via Albertoni, 15Malpighi di Bologna<br>Bologna, ITALY 40138
Comitato Etico Val Padana<br>Viale Concordia, 1<br>Cremona, ITALY 26100
**Poland**
Komisja Bioetyczna Wojskowego Instytutu Medycznego<br>ul. Szaserow 128<br>Warszawa, POLAND 04-141
**Slovakia**
Fakultna nemocnica s poliklinikou<br>Eticka komisia<br>Vojtecha Spanyola 43<br>Zilina, SLOVAKIA 012 07
Eticka komisia, Univerzitna nemocnica Bratislava<br>Pracovisko: Nemocnica akademika L. Derera Limbova 5<br>Bratislava, SLOVAKIA 833 05
**Spain**
Comite Etico de Investigacion Clinica<br>Hospital Clinico i Provincial de Barcelona<br>C/ VILLARROEL, 170<br>BARCELONA, BARCELONA SPAIN 8036
**Switzerland**
Kantonale Ethikkommission Bern (KEK)<br>Postfach 56<br>Bern, SWITZERLAND 3010
**Taiwan**
Institutional Review Board of Taipei Veterans General Hospital<br>201, Sec. 2. Shih-Pai Road<br>Taipei, TAIWAN 112
**United States**
The University of North Carolina at Chapel Hill<br>Medical School Bldg 52 105 Mason Farm Road<br>Office of Human Research Ethics CB 7097<br>Chapel Hill, NC UNITED STATES 27599-7097
Western Institutional Review Board<br>3535 Seventh Ave SW<br>Olympia, WA UNITED STATES 98502

Supplementary Fig. 1 Complete profile of cell types significantly associated with DFS . Longer DFS (red), shorter DFS (blue), and non-significant (white) are shown. Each cell type score (xCell) was stratified by >median or ≤median for the overall cohort, sunitinib arm, or placebo arm. The log2(HR) was clustered in a heatmap.

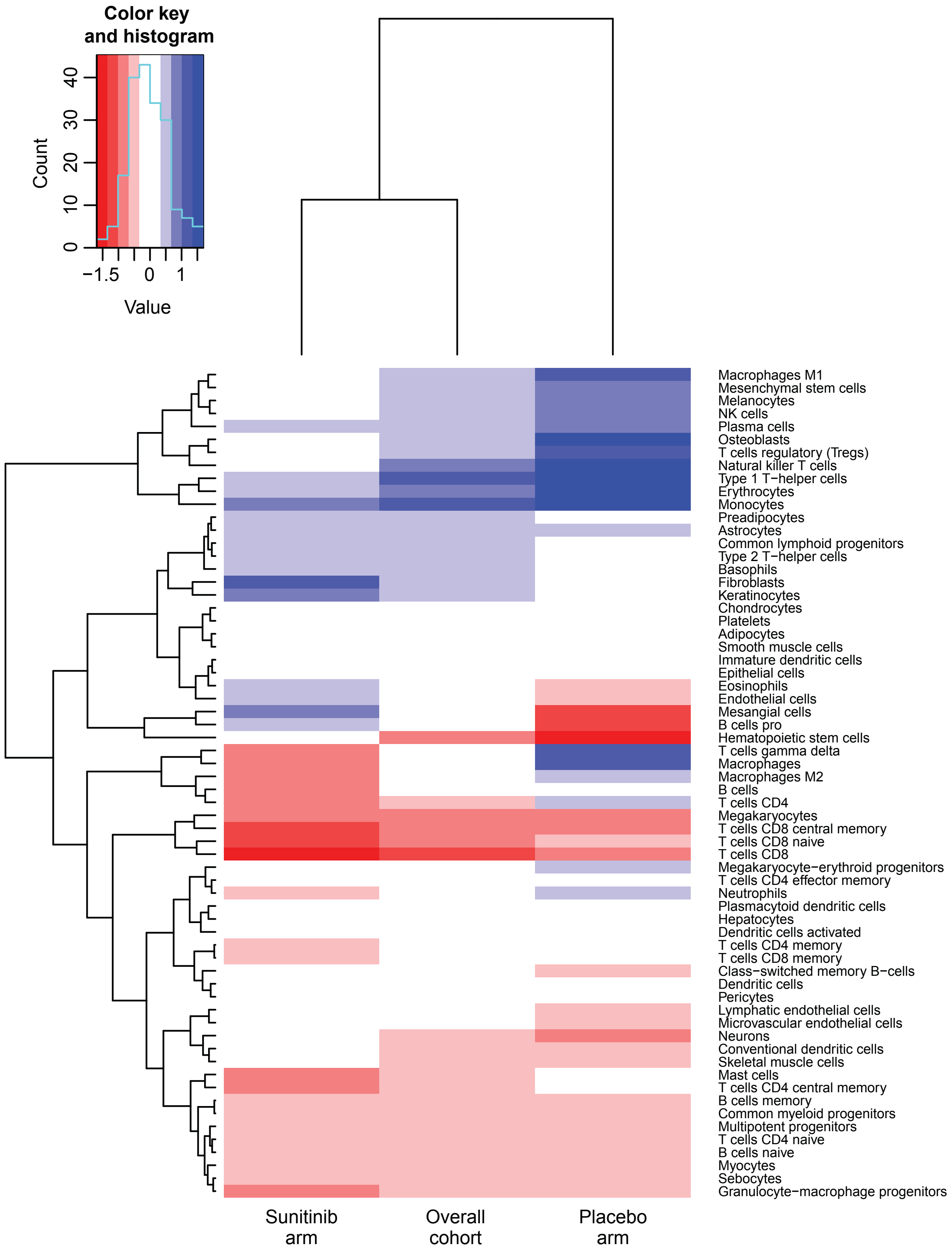

DFS=disease-free survival; HR=hazard ratio. For significant and non-significant cell types for both arms and overall cohort, data are available in raw data file FigureS1_cell_types_assoc_with_DFS/data.tsv

Supplementary Fig. 2 Identification of new transcriptomic STRAC14 signature associated with high risk of recurrence and poor prognosis. (**a**) Discovery of the STRAC14 GES according to bootstrapping frequency [derived from the placebo-treated S-TRAC trial population]. Kaplan–Meier plot of (**b**) differential DFS probability by STRAC14 in the S-TRAC overall population and (**c**) by treatment arm. Kaplan-Meier plot of (**d**) differential PFS probability, and (**e**) and differential OS probability by STRAC14 in TCGA data set and differential PFS probability by STRAC14 in the JAVELIN Renal 101 (**f**) overall data set and (**g**) by treatment arm. For panels 2b-g, a two-sided log-rank test was used and no adjustments were made for multiple comparisons.

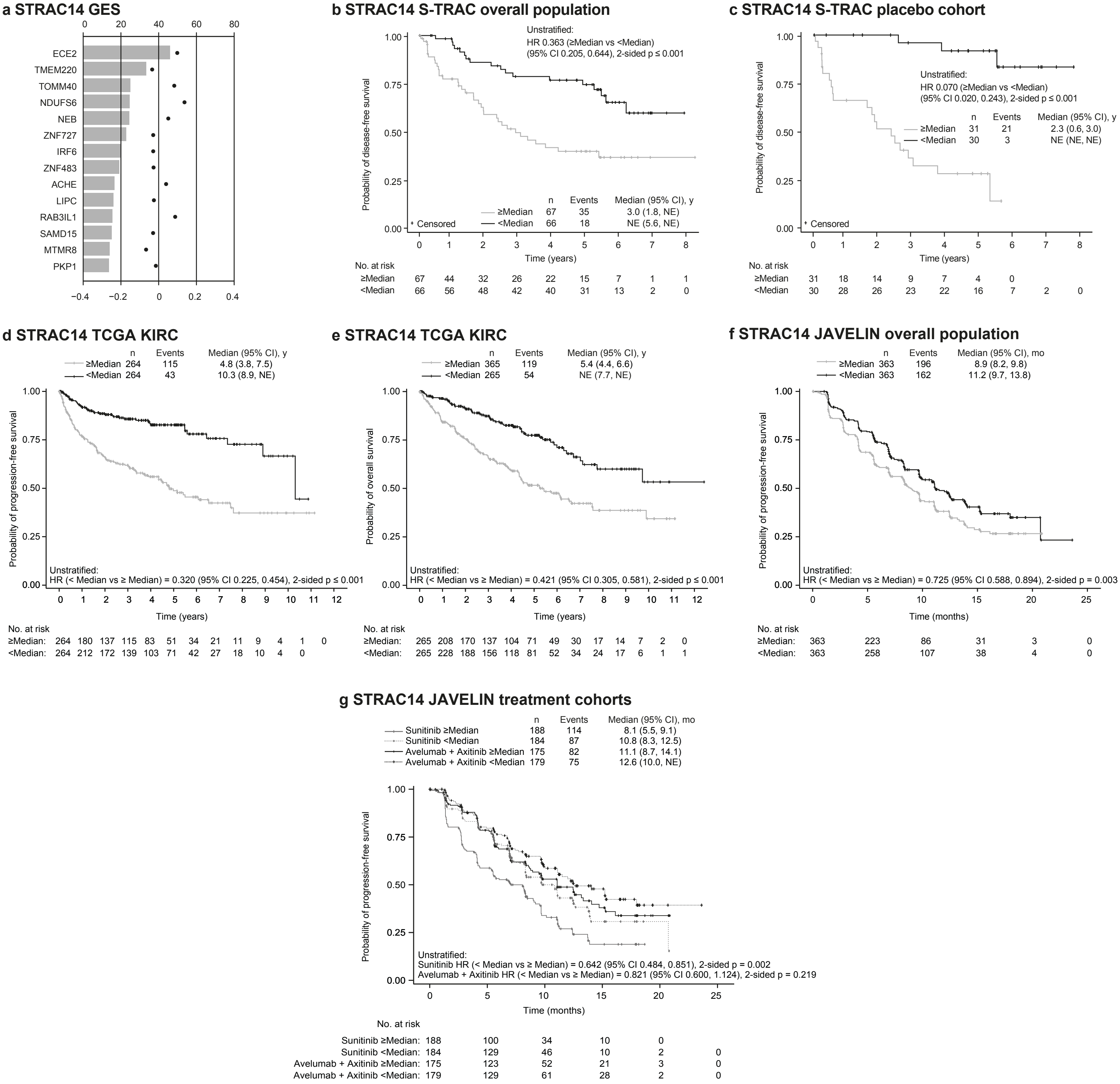

ACHE=acetylcholinesterase; CI=confidence interval; DFS=disease-free survival; ECE2=endothelin converting enzyme 2; HR=hazard ratio; IRF6= interferon regulatory factor 6; LIPC=lipase C (hepatic type); MTMR8=myotubularin related protein 8; NE=not estimable; NEB=nebulin; NDUFS6= NADH:ubiquinone oxidoreductase subunit S6; OS=overall survival; PFS=progression-free survival; PKP1=plakophilin 1; RAB3IL1=RAB3A interacting protein like 1; SAMD15=sterile alpha motif domain containing 15; STRAC14=gene expression signature of 14 genes; TCGA KIRC=The Cancer Genome Atlas Kidney Renal Clear Cell Carcinoma; TMEM220=transmembrane protein 220; TOMM40=translocase of outer mitochondrial membrane 40; ZNF483=zinc finger protein 483; ZNF727=zinc finger protein 727

Supplementary Fig. 3 Identification of new transcriptomic STRAC13 signature associated with high risk of recurrence and poor prognosis. (**a**) Discovery of the STRAC13 GES according to bootstrapping frequency [derived from entire S-TRAC trial population]. Kaplan–Meier plot of (**b**) differential DFS probability by STRAC13 in the S-TRAC overall population and (**c**) by treatment arm. Kaplan-Meier plot of (**d**) differential PFS probability, and (**e**) and differential OS probability by STRAC13 in TCGA data set and differential PFS probability by STRAC13 in the JAVELIN Renal 101 (**f**) overall data set and (**g**) by treatment arm. For panels 3b-g, a two-sided log-rank test was used and no adjustments were made for multiple comparisons.

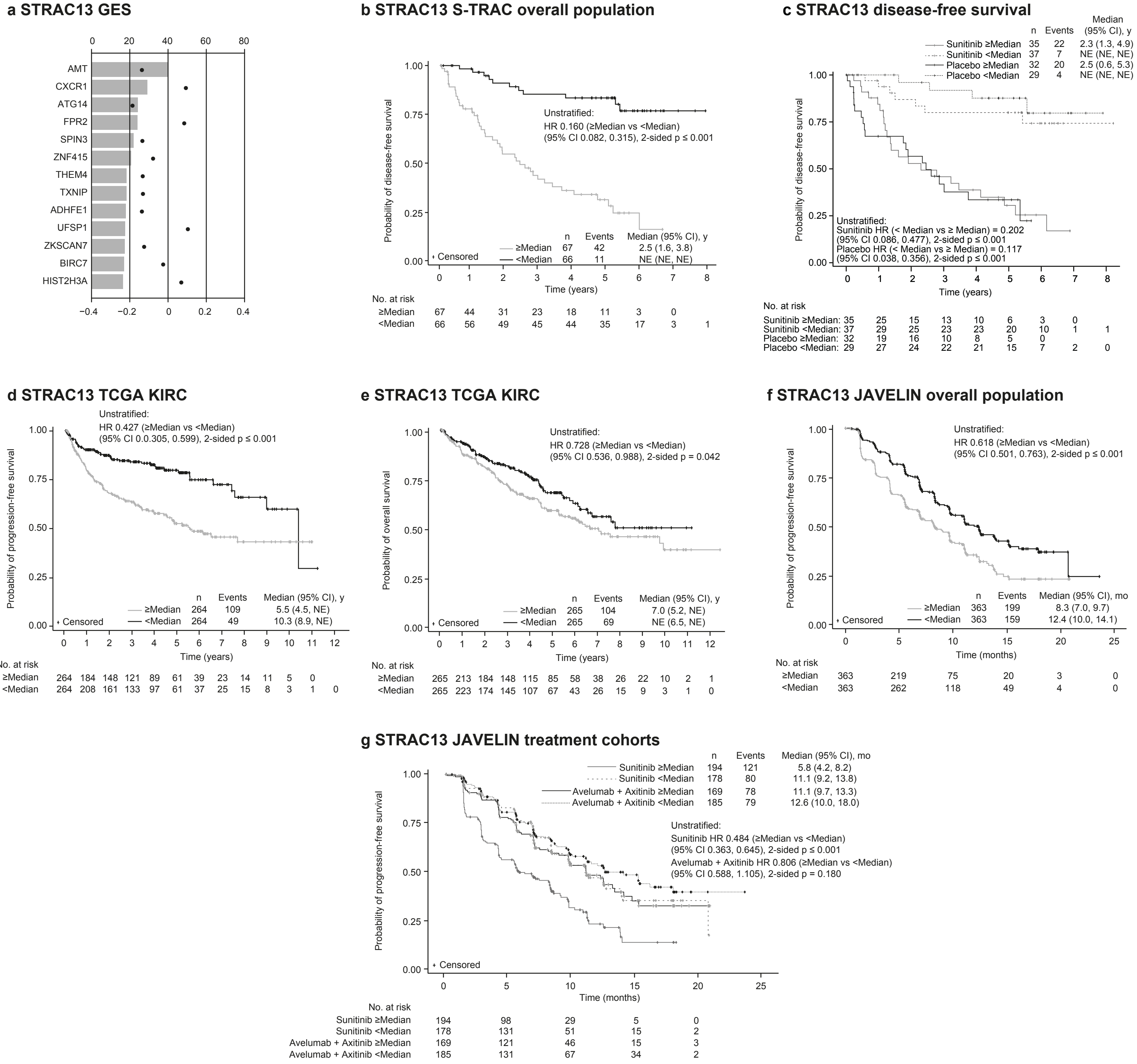

ADHFE1=alcohol dehydrogenase iron containing 1; AMT=aminomethyltransferase; ATG14=autophagy-related 14; BIRC7=baculoviral IAP repeat containing 7; CI=confidence interval; DFS=disease-free survival; CXCR1=C-X-C motif chemokine receptor 1; DFS=disease-free survival; FPR2=formyl peptide receptor 2; GES=gene expression signature; HIST2H3A=histone cluster 2 H3A; HR=hazard ratio; NE=not estimable; OS=overall survival; PFS=progression-free survival; SPIN3=spindlin family member 3; STRAC13=gene expression signature of 13 genes; TCGA KIRC=The Cancer Genome Atlas Kidney Renal Clear Cell Carcinoma; THEM4=thioesterase
