# Supplementary material for: Molecular characterization of renal cell carcinoma tumors from a phase III anti-angiogenic adjuvant therapy trial
Source: Nat Commun. 2022 Oct 10;13:5959. doi: 10.1038/s41467-022-33555-8 (PMC9550765; doi:10.1038/s41467-022-33555-8)
Supplement: Supplementary file 3 — Reporting Summary [file 41467_2022_33555_MOESM3_ESM.pdf]

nature portfolio

Corresponding author(s): Dr Motzer

Last updated by author(s): 21/6/22

Reporting Summary

Nature Portfolio wishes to improve the reproducibility of the work that we publish. This form provides structure for consistency and transparency in reporting. For further information on Nature Portfolio policies, see our Editorial Policies and the Editorial Policy Checklist.

Statistics

For all statistical analyses, confirm that the following items are present in the figure legend, table legend, main text, or Methods section.

n/a	Confirmed	
☐	☒	The exact sample size (n) for each experimental group/condition, given as a discrete number and unit of measurement
☐	☒	A statement on whether measurements were taken from distinct samples or whether the same sample was measured repeatedly
☐	☒	The statistical test(s) used AND whether they are one- or two-sided<br>*Only common tests should be described solely by name; describe more complex techniques in the Methods section.*
☐	☒	A description of all covariates tested
☐	☒	A description of any assumptions or corrections, such as tests of normality and adjustment for multiple comparisons
☐	☒	A full description of the statistical parameters including central tendency (e.g. means) or other basic estimates (e.g. regression coefficient) AND variation (e.g. standard deviation) or associated estimates of uncertainty (e.g. confidence intervals)
☐	☒	For null hypothesis testing, the test statistic (e.g. F, t, r) with confidence intervals, effect sizes, degrees of freedom and P value noted<br>*Give P values as exact values whenever suitable.*
☒	☐	For Bayesian analysis, information on the choice of priors and Markov chain Monte Carlo settings
☒	☐	For hierarchical and complex designs, identification of the appropriate level for tests and full reporting of outcomes
☒	☐	Estimates of effect sizes (e.g. Cohen's d, Pearson's r), indicating how they were calculated

Our web collection on statistics for biologists contains articles on many of the points above.

Software and code

Policy information about availability of computer code

Data collection	Javelin RENAL 101: https://doi.org/10.1038/s41591-020-1044-8; TCGA: https://gdc.cancer.gov/<br>Oracle Clinical was used to collect the clinical data from S-TRAC.
Data analysis	For the WES NGS processing, the vendor's (Personalis, https://www.personalis.com/biomarker-discovery/) analysis pipelines were utilized. D4C software-Biomedical Intelligence Cloud. Data4Cure, Inc. La Jolla, CA, United States, 2019 (https://www.data4cure.com) was used for integrated marker analyses.<br>Databases used in this analysis included TCGA (https://doi.org/10.1038/nature12222) and MSigDB (https://doi. org/10.1093/bioinformatics/btr260).

For manuscripts utilizing custom algorithms or software that are central to the research but not yet described in published literature, software must be made available to editors and reviewers. We strongly encourage code deposition in a community repository (e.g. GitHub). See the Nature Portfolio guidelines for submitting code & software for further information.

Data

Policy information about availability of data

All manuscripts must include a data availability statement. This statement should provide the following information, where applicable:

- Accession codes, unique identifiers, or web links for publicly available datasets
- A description of any restrictions on data availability
- For clinical datasets or third party data, please ensure that the statement adheres to our policy

Subject to certain criteria, conditions and exceptions, Pfizer will also provide access to related individual anonymized participant data. See https://www.pfizer.com/science/clinical-trials/ trial-data-and-results/ for more information. Source data are provided with this paper. RNA-seq somatic alterations, and clinical outcomes data supporting the findings of this manuscript are deposited in Source data file.

Field-specific reporting

Please select the one below that is the best fit for your research. If you are not sure, read the appropriate sections before making your selection.

☒ Life sciences ☐ Behavioural & social sciences ☐ Ecological, evolutionary & environmental sciences

For a reference copy of the document with all sections, see nature.com/documents/nr-reporting-summary-flat.pdf

Life sciences study design

All studies must disclose on these points even when the disclosure is negative.

Sample size	Previously described for S-TRAC in Ravaud et al. N Engl J Med 2016; 375:2246-2254. In S-TRAC, it was determined that '320 events of recurrence, diagnosis of a second cancer, or death would provide a power of 90% to detect a hazard ratio of 0.69 for the comparison between sunitinib and placebo at a two-sided significance level of 0.05. Since there was a lower-than-expected rate of disease-free survival during the trial, the protocol was amended to specify that the final analysis would occur approximately 5 years after the last patient underwent randomization. It was estimated that approximately 258 events would have occurred at that time, which would provide a power of 84% to detect statistical significance for a hazard ratio of 0.69.'<br><br>The size of the S-TRAC subpopulation with available tumor tissue was limited to those patients who provided consent for exploratory analyses.
Data exclusions	Of the 615 patients in the intent-to-treat population in the S-TRAC trial, tumor tissue from nephrectomy or tumor biopsy were available for 171 (27.8%) patients (91 sunitinib, 80 placebo) for the WES analysis, and 133 (21.6%) patients (72 sunitinib, 61 placebo) for the GES analysis. This breaks down as follows. Anonymized archival tumor tissue from nephrectomy or tumor biopsy (FFPE block or slides) were collected on an optional basis for 212 patients who properly consented for exploratory analysis. Following the prior tumor tissue analyses, 193 individual specimens were available for molecular profiling, of which 171 (27.8%) (sunitinib, n = 91; placebo, n = 80) returned results for the WES analysis, and 133 (21.6%) (sunitinib, n = 72; placebo, n = 61) returned results for the GES analysis; 11 cases fail both DNA and RNA extractions, while another 11 fail the DNA extraction and/or did not pass QC (at the library or sequencing steps) and similarly 51 fail the RNA extraction and/or QC steps.
Replication	For elastic net analysis of S-TRAC gene signatures: For each dataset, we performed 1000 bootstrap runs of fitting a Cox proportional-hazards model for DFS with regularization by an elastic net penalty and a fivefold cross-validation. The STRAC14, STRAC13, and STRAC11 GES were further verified using two independent datasets, the JAVELIN Renal 101 study in patients with metastatic advanced RCC (n=720) and the KIRC dataset of TCGA (n=488)
Randomization	Previously described in Ravaud et al. N Engl J Med 2016; 375:2246-2254. In S-TRAC, 'randomization was stratified according to the UISS-defined high-risk group, the ECOG score (<2 vs. 2), and country of residence. Patients were assigned in a 1:1 ratio to receive either oral sunitinib (50 mg per day) or placebo on a 4-weeks-on, 2-weeks-off schedule for 1 year. Dose interruptions or dose reductions to 37.5 mg per day were allowed, depending on the type and severity of toxicity. Treatment continued until disease recurrence, diagnosis of a secondary cancer, unacceptable toxic effects, or consent withdrawal.
Blinding	Blinding was not relevant for this analysis. Blinding methods are published for each of the available data sets used in this analysis.

Reporting for specific materials, systems and methods

We require information from authors about some types of materials, experimental systems and methods used in many studies. Here, indicate whether each material, system or method listed is relevant to your study. If you are not sure if a list item applies to your research, read the appropriate section before selecting a response.

Materials & experimental systems

n/a	Involved in the study
☒	☐ Antibodies
☒	☐ Eukaryotic cell lines
☒	☐ Palaeontology and archaeology
☒	☐ Animals and other organisms
☐	☒ Human research participants
☐	☒ Clinical data
☒	☐ Dual use research of concern

Methods

n/a	Involved in the study
☒	☐ ChIP-seq
☒	☐ Flow cytometry
☒	☐ MRI-based neuroimaging

Human research participants

Policy information about studies involving human research participants

Population characteristics

In the WES analysis, patients who received sunitinib (n=91) were predominantly aged <65 years (73.6%, n=67), of white ethnicity (83.5%, n=76) with an ECOG PS of 0 (71.4%, n=65). Corresponding values for patients who received placebo (n=80) in the WES analysis were 65% (n=52), 87.5% (n=70), and 73.8% (n=59), respectively.
For the GES analysis, patients who received sunitinib (n=72) were predominantly aged <65 years (73.6%, n=53), of white ethnicity (80.6%, n=58) with an ECOG PS of 0 (69.4%, n=50). Corresponding values for patients who received placebo (n=61) in the GES analysis were 70.5% (n=43), 86.9% (n=53), and 65.6% (n=40), respectively.

Recruitment

From September 19, 2007, to April 7, 2011, 615 patients at 99 centers in 21 countries were enrolled in the prospective, randomized, double-blind, S-TRAC phase 3 trial. Additional consent was required for the optional WES/GES exploratory analysis.

Ethics oversight

The S-TRAC trial was approved by the independent review board or ethics committee at each center, and was conducted in accordance with International Conference on Harmonization Good Clinical Practice guidelines. All the patients provided written informed consent

Note that full information on the approval of the study protocol must also be provided in the manuscript.

Clinical data

Policy information about clinical studies

All manuscripts should comply with the ICMJE guidelines for publication of clinical research and a completed CONSORT checklist must be included with all submissions.

Clinical trial registration

S-TRAC: NCT00375674

Study protocol

S-TRAC previously described in Ravaud et al. N Engl J Med 2016; 375:2246-2254

Data collection

S-TRAC previously described in Ravaud et al. N Engl J Med 2016; 375:2246-2254. Briefly, From September 19, 2007, to April 7, 2011, 615 patients were enrolled at 99 centers in 21 countries.

Outcomes

S-TRAC previously described in Ravaud et al. N Engl J Med 2016. 'The primary end point was the duration of disease-free survival, which was defined as the interval between randomization and the first tumor recurrence, the occurrence of metastasis or a secondary cancer (as assessed by blinded independent central review), or death. Secondary end points included overall survival, safety, and health-related quality of life.'
